# Human Sialidase Neu3 is S-Acylated and Behaves Like an Integral Membrane Protein

**DOI:** 10.1038/s41598-017-04488-w

**Published:** 2017-06-23

**Authors:** Macarena Rodriguez-Walker, Jose L. Daniotti

**Affiliations:** Centro de Investigaciones en Química Biológica de Córdoba, CIQUIBIC, CONICET and Departamento de Química Biológica Ranwel Caputto, Facultad de Ciencias Químicas, Universidad Nacional de Córdoba, Ciudad Universitaria, Córdoba, X5000HUA Argentina

## Abstract

Membrane-bound sialidase Neu3 is involved in the catabolism of glycoconjugates, and plays crucial roles in numerous biological processes. Since the mechanism of its association with membranes is still not completely understood, the aim of this work was to provide further information regarding this aspect. Human Neu3 was found to be associated with the plasma membrane and endomembranes, and it was not released from the lipid bilayer under conditions that typically release peripheral membrane proteins. By different experimental approaches, we demonstrated that its C-terminus is exposed to the cytosol while another portion of the protein is exposed to the extracellular space, suggesting that Neu3 possesses the features of a transmembrane protein. However, in silico analysis and homology modeling predicted that the sialidase does not contain any α-helical transmembrane segment and shares the same β-propeller fold typical of viral and bacterial sialidases. Additionally, we found that Neu3 is S-acylated. Since this post-translational modification is restricted to the cytosolic side of membranes, this finding strongly supports the idea that Neu3 may contain a cytosolic-exposed domain. Although it remains to be determined exactly how this sialidase crosses the lipid bilayer, this study provides new insights about membrane association and topology of Neu3.

## Introduction

Sialidases are a family of glycohydrolytic enzymes that catalyze the removal of sialic acid residues from glycoproteins, oligosaccharides and glycolipids. They are widely distributed in vertebrates and also in a variety of viruses, bacteria and parasites, with the four types of mammalian sialidases identified to date having been designated Neu1, Neu2, Neu3 and Neu4. They are encoded by four different genes and differ in their subcellular localization, enzymatic properties and substrate specificity. A molecular and biochemical description of these mammalian sialidases forms as well as their functional implications has been reviewed elsewhere^1, 2^. In particular, Neu3 was first identified as being a plasma membrane-associated sialidase specific for gangliosides^3^, and in recent years, it has been reported to be involved in numerous biological pathways, including cell adhesion, migration, differentiation, cancer progression, apoptosis and endocytosis^4–9^. In this sense, Neu3 plays a crucial role in the regulation of transmembrane signaling in a dual way: through the modulation of ganglioside catabolism and by direct interaction with signaling molecules such as the Epidermal Growth Factor Receptor (EGFR), caveolin-1 (Cav-1), Grb2 and Integrin β4^7, 10–13^. Interestingly, it was proposed that glycoproteins may be also among the physiological substrates of Neu3^14^. In fact, it has been recently demonstrated that Neu3 can directly desialylate EGFR glycoprotein and in this way, modulates its activation^15^. In addition, although Neu3 is generally thought to be mainly localized at the plasma membrane, other laboratories and ours have reported that Neu3 is also present in membranes from endosomal compartments^9, 16^, where it interacts with key proteins involved in protein folding and intracellular trafficking^17^.

Despite the fact that it is widely accepted that Neu3 is membrane-bound and that it interacts strongly with membranes, a complete understanding of the mechanism of its association with cellular membranes is still lacking, and its topology also remains a mystery. In 1999, Miyagi *et al*. proposed that Neu3 was a type I integral membrane protein with its C-terminus facing the cytosol, and with a membrane-binding domain near the center of the polypeptide^3^. Later, Chavas *et al*. reported the first high resolution x-ray structure of a mammalian sialidase: the human cytosolic Neu2^18^. This structure shows the typical β-propeller fold that has been already described for bacterial and viral sialidases^19, 20^, indicating that despite the low sequence identity between viral, bacterial and mammalian sialidases, they have a common folding topology, with active site residues highly conserved among species. To date, however, no crystallographic studies of Neu3 have been reported, probably because of its hydrophobic nature making it difficult to obtain a crystal structure. In the absence of crystallographic data, homology models of the remaining three members of the mammalian sialidase family have been developed based on the crystal structure of Neu2^21^, with Neu3 being predicted to have the typical structure of sialidases: a β-propeller composed of four anti-parallel β-sheets organized in six blades. Analysis of the Neu3 homology model ruled out the possibility of it being a type I transmembrane protein, since if this were the case, the highly conserved amino acid residues involved in the active site formation would be separated by the lipid bilayer. In the search of alternative models, in 2007, Zanchetti *et al*. suggested that Neu3 behaves as a peripheral membrane protein associated with the outer leaflet of the plasma membrane^16^. However, this topological distribution of Neu3 is not fully consistent with the results recently obtained by other laboratories and ours, mainly concerning Neu3 sialidase protein interactors^11, 12, 17^, post-translational modifications (this work) and regulation of clathrin-mediated endocytosis^9^. In the present study, we provide a comprehensive analysis of Neu3 membrane topology and propose an alternative view of its association with membranes.

## Results

### Expression of human sialidase Neu3 in CHO-K1 cells

**Figure 1:**
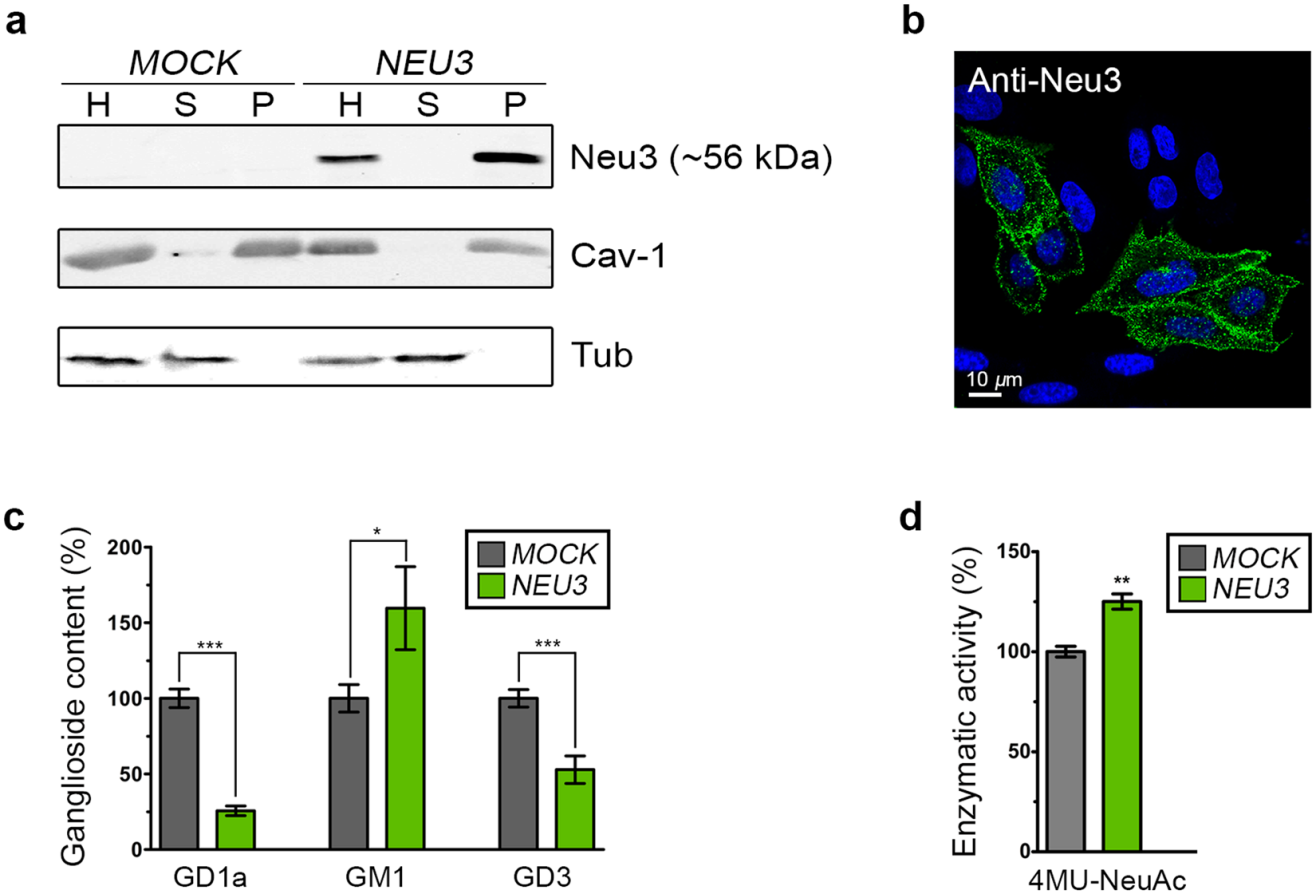
Expression of human sialidase Neu3 in CHO-K1 cells. (**a**) *MOCK*- and *NEU3*-transfected cells were mechanically lysed, and the homogenates (H) were ultracentrifuged. Neu3 expression in both supernatant (S) and pellet (P) fractions was determined by Western blotting using anti-Neu3 antibody. Caveolin-1 (Cav-1) and tubulin (Tub) were used as endogenous markers of a membrane-bound and a soluble protein, respectively. (**b**) Fixed and permeabilized *NEU3*-transfected cells were immunostained with antibody against Neu3 and visualized by confocal microscopy. Cells nuclei were stained blue with Hoechst dye. (**c**) Ganglioside content of *MOCK*- and *NEU3*-transfected cells. Cells were incubated with antibody against GD3, antibody against GD1a or CTx*β* (which binds to GM1) at 4 °C for 60 min to label the plasma membrane-associated gangliosides. Then, cells were fixed, permeabilized, incubated with antibody against Neu3 and visualized by confocal microscopy. Ganglioside content was quantified according to its fluorescence intensity using ImageJ software. Values are related to the percentage of each ganglioside in *MOCK*-transfected cells and presented as mean ± S.E.M. Statistical analyses were made using Student’s t test with significance being attributed at the 95% level of confidence (*P ≤ 0.05; **P ≤ 0,01; ***P ≤ 0,001). (**d**) Total sialidase activity in cell homogenates of *MOCK*- and *NEU3*-transfected cells towards the artificial substrate 4MU-NeuAc.

In order to characterize the membrane topology of sialidase Neu3, and after several failed attempts to detect the endogenous protein of CHO-K1 and HeLa cells with available anti-Neu3 antibodies, we decided to transiently transfect CHO-K1 cells with a full-length human Neu3 tagged at the C-terminus with a c-Myc epitope (hereinafter referred to as Neu3). Homogenates obtained from *NEU3*-transfected cells analyzed by Western blotting with anti-Neu3 antibody, showed Neu3 migrating with an apparent molecular mass of ∼56 kDa (Fig. 1a), whereas no immunostained band was observed in homogenates from *MOCK*-transfected CHO-K1 cells. Subcellular fractionation of the total cell homogenate into soluble and rough particulate cell compartments was carried out by ultracentrifugation, with more than 98% of the expressed sialidase being recovered in the particulate fraction, thereby clearly demonstrating its membrane association (Fig. 1a). The endogenous protein markers caveolin-1 (Cav-1) and tubulin (Tub) were run simultaneously and used as controls of a membrane-associated and a soluble protein, respectively. The subcellular distribution of Neu3 in CHO-K1 cells visualized by confocal microscopy included a pool at the cell surface, as expected, and another pool at endomembranes (Fig. 1b), previously identified as membranes from recycling and early endosomes (Rab11 and Rab5 positive compartments)^9^. Next, we evaluated if the expressed sialidase was able to modify the ganglioside content of cells, and since gangliosides GD3 and GD1a can be easily detected with specific monoclonal antibodies, we quantified the amount of these Neu3 substrates by immunofluorescence in CHO-K1 cells modified to express the ganglioside of interest^22^. As shown in Fig. 1c, Neu3 expression resulted in a clear and significant decrease in the GD3 and GD1a content, with a concomitant increase in the content of GM1, whose inner sialic acid residue is not accessible for Neu3. These results are in line with previous reports showing substrate specificity for Neu3^3, 23^. Additionally, in *NEU3*-transfected cells, the total sialidase activity was found to be 25% higher than in *MOCK*-transfected cells using the artificial substrate 4MU-NeuAc (Fig. 1d). Taken together, results from these experiments indicate that the expressed sialidase had the expected subcellular distribution and was enzymatically active both *in vivo* and *in vitro*.

### Neu3 behaves as an integral membrane protein

**Figure 2:**
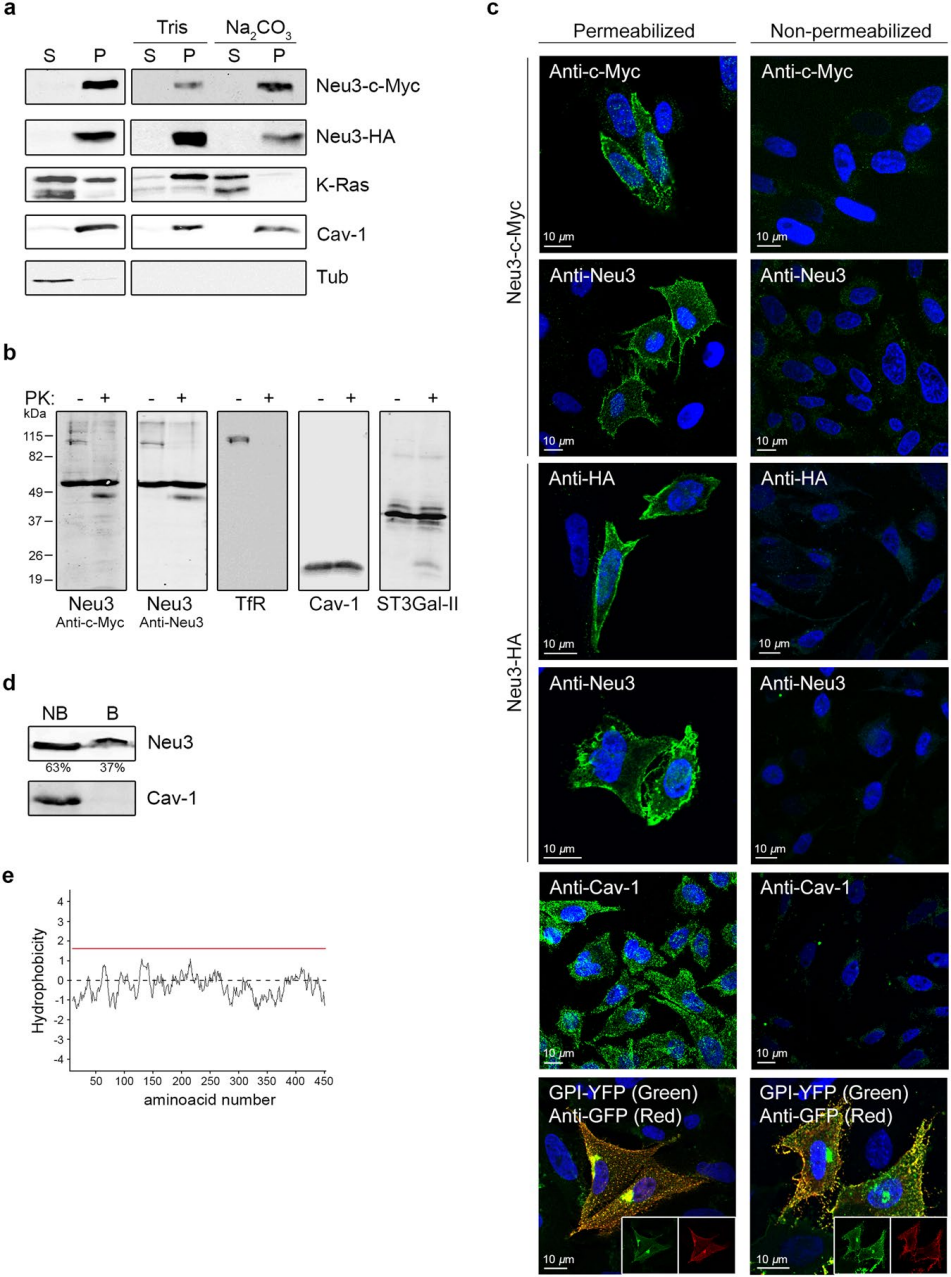
Neu3 behaves as an integral membrane protein. (**a**) Soluble (S, first lane) and membrane (P, second lane) fractions of *NEU3*-transfected cells were obtained as described in Fig. 1a. The latter fraction was then incubated for 40 min on ice with Tris buffer (control) or Na_2_CO_3_ at pH 11.5 in order to extract peripheral membrane proteins. After treatment, membranes were collected by ultracentrifugation, and membrane-bound (P, fourth and sixth lanes) and solubilized (S, third and fifth lanes) proteins were analyzed by Western blotting using anti-Neu3 antibody. K-Ras and Cav-1 were used as markers of an extractable and a non-extractable membrane protein, respectively. Tub was used as a marker of a soluble protein. (**b**) Intact *NEU3*-transfected cells were treated with (+) or without (−) 100 µg/ml proteinase K (PK) for 30 min at 37 °C. The remaining proteins were run on a SDS-PAGE gel and Western blotted with anti-c-Myc and anti-Neu3 antibodies. Transferrin Receptor (TfR) was used as a marker of a PK-accessible protein, whereas Cav-1 and sialyltransferase ST3Gal-II were used as markers of proteins not accessible to PK. The molecular masses in kDa of the standard proteins are shown on the left. (**c**) Antibody accessibility in permeabilized and non-permeabilized cells. The signal of the indicated antibodies were analyzed after fixation in cells treated with 0.1% saponin in PBS/BSA (permeabilized) or with PBS/BSA alone (non-permeabilized). Cav-1 was used as a marker of an inner membrane protein detected only after cell permeabilization, and GPI-YFP was used as a marker of an outer membrane protein accessible by antibody without permeabilization. Cells nuclei were stained blue with Hoechst dye. (**d**) Cell surface proteins exposed to the extracellular environment of *NEU3*-transfected cells were biotinylated and isolated by streptavidin agarose pull-down. Biotinylated (B) and non-biotinylated (NB) fractions were analyzed by Western blotting. Endogenous marker Cav-1 was used as control of a membrane protein not exposed to the extracellular environment. Densitometric analysis indicating the percentage of Neu3 in each fraction is shown. (**e**) Kyte-Doolittle hydrophobicity plot for Neu3 sequence using a window size of 19. The red line corresponds to a hydrophobicity score of 1.6 and designates an empirical cutoff value for α-helix transmembrane segments of ~19 amino acids long.

As mentioned in the introduction, it has been previously proposed that Neu3 behaves as a peripheral membrane protein^16^. Dissociation of peripheral proteins from membranes can be achieved through treatment with solutions of high pH or high salt concentration, while in the same experimental conditions, integral membrane proteins remain bound to the membrane fraction. Therefore, sodium carbonate extraction at pH 11.5 was performed to determine whether membrane-bound Neu3 could be recovered in the soluble fraction. Exposure of membranes to alkaline conditions resulted in almost a complete solubilization of K-Ras (Fig. 2a, third row), a protein whose membrane binding is mostly driven by electrostatic interactions between a polybasic region in the protein and negatively charged membranes^24^. In sharp contrast, Neu3 (c-Myc epitope tagged) was not significantly solubilized under the same conditions, and in fact remained almost completely in the membrane fraction (Fig. 2a, first row). A similar behavior was also observed for the human Neu3 containing a C-terminal HA tag, precluding the possibility that the addition of the epitope could modify membrane association of the sialidase (Fig. 2a, second row). As expected, the integral membrane protein Cav-1 was not solubilized by alkaline treatment (Fig. 2a, fourth row). Incubation of membranes with Tris buffer as control had no significant effect on the association of the analyzed proteins with the membranes. Furthermore, we were unable to find any condition that allowed extraction of Neu3 from membranes, not even exposure to high salt concentrations -up to 1.5 M NaCl- (results not shown). Overall, these results suggest that Neu3 has the features of an integral membrane protein.

In order to determine the membrane orientation of the C-terminus of Neu3, protease protection assays were carried out in intact cells using proteinase K (PK), a non-specific protease that in Neu3 sequence has more than 200 cleavage sites. As mentioned in Methods, intact cells grown in plates were incubated with PK for 30 min at 37 °C. Then, cells were washed, collected, and the products analyzed by Western blotting. As expected, the transferrin receptor (TfR) which is mainly exposed to the extracellular environment was completely degraded by PK, whereas the Golgi membrane resident protein ST3Gal-II and the plasma membrane Cav-1 were not accessible to PK (Fig. 2b). It is worth mentioning that Cav-1 is a ~22 kDa integral membrane protein that do not span the lipid bilayer completely and instead is inserted into the inner leaflet of the plasma membrane adopting a hairpin-like structure, with both the N- and C- termini facing the cytoplasm^25^. These control experiments indicated that PK was only able to degrade proteins present in the plasma membrane and exposed to the extracellular space. In the case of Neu3, this treatment led to the generation of two protease-protected fragments using an antibody directed to the c-Myc epitope (Fig. 2b). One of the fragments corresponded to the full-length version of the enzyme (~56 kDa), and was consistent with the fact that Neu3 is present in endomembranes and clearly not accessible to PK over the time-course of the experiment. The other PK-protected fragment had ~48 kDa and might correspond to the sialidase lacking ~70 amino acids of its N-terminus. Exactly the same result was obtained using an anti-Neu3 antibody (Fig. 2b). Unfortunately, there is no information about the precise epitope recognized by this commercial antibody, however, given our results, we expect it to be near the C-terminus of the protein. From these experiments, we conclude that Neu3 at the plasma membrane has a portion exposed to the extracellular space being accessible to PK, and that the C-terminus of the protein is somehow protected, leading us to speculate that it might be exposed to the cytosolic side of the membrane.

We then performed immunostaining of permeabilized and non-permeabilized cells using anti-c-Myc antibody. The signal of the antibody was observed as expected when cells were permeabilized with 0.1% saponin. However, the signal was hardly detectable in non-permeabilized cells (Fig. 2c, first row). Thus, although the protein itself was at the plasma membrane, the c-Myc epitope was not accessible to the antibody from the exterior of the cell. Similar results were observed using anti-Neu3 antibody (Fig. 2c, second row). In the same way, detection of Neu3 tagged at the C-terminus with a HA epitope by anti-HA and anti-Neu3 antibodies could only be achieved in permeabilized cells (Fig. 2c, third and fourth rows). Likewise, Cav-1 was stained only when cells were permeabilized (Fig. 2c, fifth row). As a control of a protein exposed to the extracellular space, we transfected cells with GPI-YFP, a fusion protein containing the total sequence of YFP fused to a glycosylphosphatidylinositol (GPI)-membrane attachment signal. The subcellular distribution of GPI-YFP in CHO-K1 cells has been already described by our group^22^, and included a pool at the cell surface and another pool at the Golgi complex. When cells were permeabilized and incubated with antibody to GFP, we observed a perfect colocalization in both pools between YFP fluorescence and the antibody signal. In contrast, in non-permeabilized cells, the antibody to GFP only gave rise to a cell surface staining, although the YFP fluorescence could still be seen in the Golgi complex (Fig. 2c, sixth row). Taken together, results from both the PK and the immunofluorescence experiments showed that the C-terminus of Neu3 is somehow protected and inaccessible from the exterior of the cell. Therefore, this extremity of Neu3 seems to be oriented towards the cytosol and protected by the lipid bilayer. The same concept might also apply to the epitope being recognized by anti-Neu3 antibody, although we do not know the precise localization of this antigenic region in human Neu3.

A selective cell-surface protein biotinylation was performed in intact cells using the membrane-impermeable EZ-Link Sulfo-NHS-SS-biotin reagent. After incubation, biotinylated (B) proteins (exposed to the extracellular space) were separated from non-biotinylated (NB) proteins using streptavidin agarose beads. As shown in Fig. 2d, most of the sialidase was recovered in the NB fraction, indicating that the main pool of the enzyme was localized intracellularly, presumably in endosomal membranes. The remaining 37% of Neu3 was accessible to biotinylation, suggesting that at the plasma membrane a portion of Neu3 was exposed to the extracellular environment, as already seen in the PK protection assays. To exclude the possibility that B fractions were contaminated by proteins coming from the inside of the cells, we analyzed the distribution of Cav-1. As expected for a plasma membrane protein not exposed to the extracellular space, Cav-1 was detected only in NB fractions (Fig. 2d). Taken together, these results prompted us to propose that Neu3 is an integral membrane protein with a portion exposed to the extracytosolic space and a portion containing at least the C-terminus exposed to the cytosol.

In order to gain additional information about this proposed membrane topology, we performed a computer analysis along the primary sequence of Neu3. A Kyte-Doolittle hydrophobicity plot of Neu3 (Fig. 2e) indicated that the protein was not likely to contain any transmembrane segment because its score stayed below the red line for all the sequence. This red line corresponds to a hydrophobicity score of 1.6 and designates an empirical cutoff value for α-helical transmembrane segments of ~19 amino acids long^26^. Furthermore, prediction of transmembrane domains using PHOBIUS (http://phobius.sbc.su.se/), TMHMM (http://www.cbs.dtu.dk/services/TMHMM/), HMMTOP (http://www.enzim.hu/hmmtop/) and PSORT II (http://psort.hgc.jp/form2.html) software did not suggest the existence of α-helical transmembrane segments, as previously reported^16, 27^. Thus, results from these analyses suggested that Neu3 is not a canonical α-helical transmembrane protein, and instead spans the lipid bilayer by a different mechanism.

### Neu3 is S-acylated

**Figure 3:**
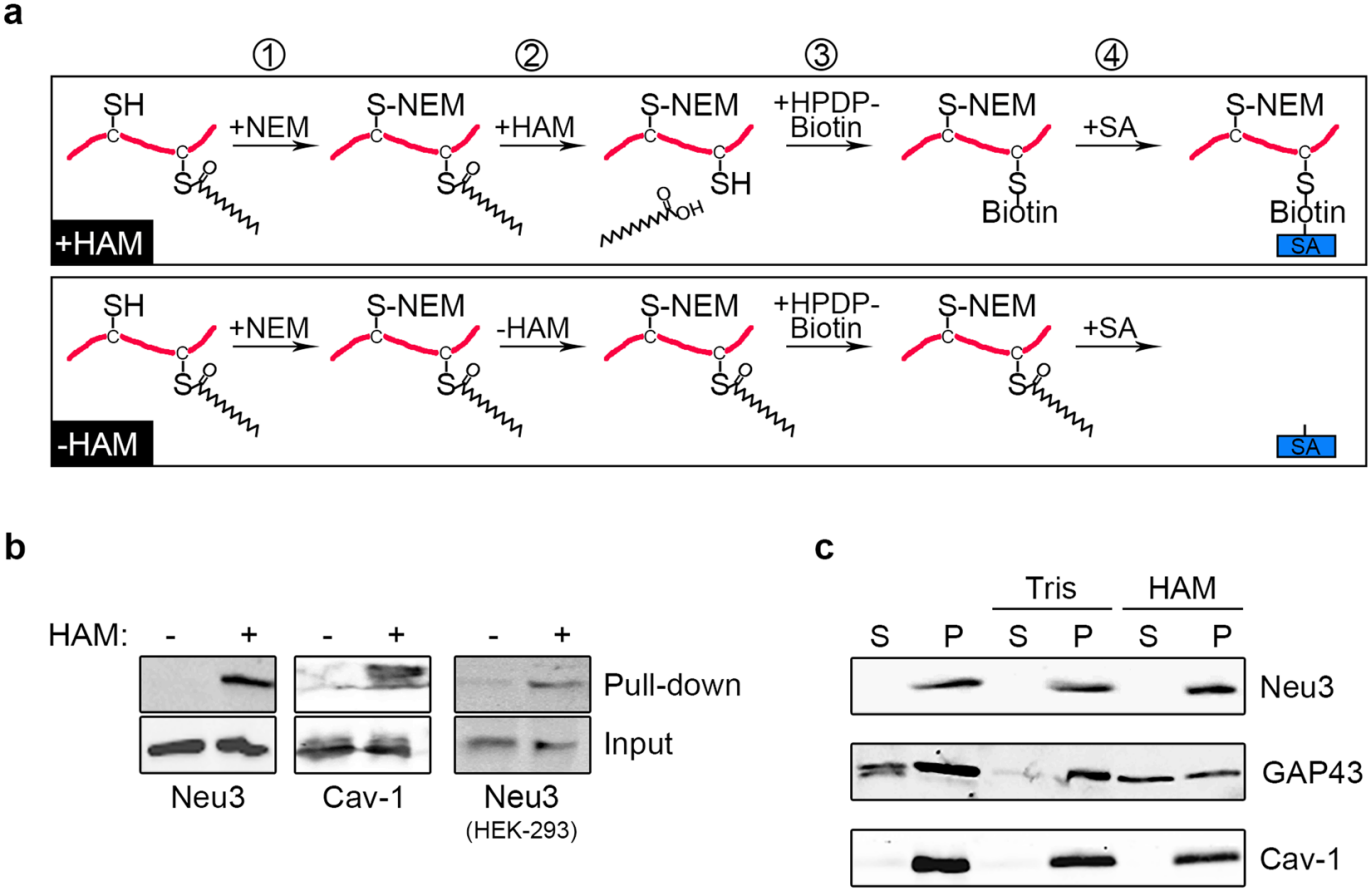
Neu3 is S-acylated. (**a**) A schematic diagram of the acyl-biotin exchange (ABE) assay. (1) Irreversible blockade of free thiol groups using N-ethylmaliemide (NEM); (2) Specific cleavage of thioester bonds and unmasking of acylated cysteines by hydroxylamine (HAM). In this step, omission of HAM served as a negative control; (3) Labeling of the S-acylated cysteines using the thiol-reactive biotinylation reagent HPDP-biotin; (4) Pull-down of the biotinylated proteins with streptavidin agarose beads (SA). (**b**) Cell lysates from *NEU3*-transfected cells were subjected to ABE analysis. For the negative control, HAM was substituted by Tris buffer (-HAM). Cav-1 was used as a marker of a known S-acylated protein. Neu3 purified from HEK-293 cells was also subjected to ABE analysis. (**c**) Effect of HAM deacylation of Neu3 on its membrane association. Membrane fractions (P, second lane) were treated with Tris buffer (control) or with HAM. After treatment, membranes were collected by ultracentrifugation, and membrane-bound (P, fourth and sixth lanes) and solubilized (S, third and fifth lanes) proteins were analyzed by Western blotting using anti-Neu3 antibody. As the two covalently-bound fatty acids of GAP43 are directly involved in its membrane association, GAP43 was used as a marker of a protein released into the supernatant after treatment with HAM. Cav-1 was used as a marker of an S-acylated protein not released after HAM treatment.

Proteins can be covalently modified with lipids through multiple mechanisms, with one of the most studied lipid modifications being S-acylation, a post-translational modification that consists in the addition of a long-chain fatty acid (mainly palmitic acid) to a cysteine residue via a thioester linkage^28^. In fact, S-acylation is a reversible post-translational modification that regulates membrane association, protein trafficking, signaling and many other biological processes. Therefore, we speculated that given the strong membrane affinity of Neu3, the attachment of fatty acids to cysteine residues of the sialidase would be an interesting chemical modification to be explored. To carry this out, cell lysates from *NEU3*-transfected cells were subjected to the acyl-biotin exchange (ABE) assay, which allows the detection of S-acylated proteins (Fig. 3a). Hydroxylamine (HAM) was used to specifically cleave the thioester linkage between a fatty acid and a cysteine, thereby allowing the specific incorporation of biotin onto the newly available thiol group. After the pull-down of biotinylated proteins of *NEU3*-transfected cells with streptavidin agarose beads, a band corresponding to Neu3 was detected in the sample treated with HAM, indicating that at least one acyl group was bound to Neu3 via a thioester linkage (Fig. 3b). In this experiment, Cav-1 was used as a control of a known acylated protein^29^. In order to demonstrate further that Neu3 is S-acylated, we performed the ABE assay using a commercially available Neu3 protein purified from human HEK-293 cells. As shown in Fig. 3b, a band of the protein was also detected in the sample treated with HAM. Given that there is no well-defined consensus sequence for S-acylation other than the requirement of a cysteine residue^30^, and taking into account that Neu3 sialidase possesses 21 cysteines, it remains to be determined which of them are acylated.

Since acylation of several peripheral proteins mediates their association with the plasma membrane, and as the removal of fatty acids causes a decreased membrane binding, we tested the effect of HAM deacylation of Neu3 on its membrane association. Membrane fractions obtained after subcellular fractionation were treated with Tris buffer or with HAM, and the distribution of Neu3 and control proteins between particulate and soluble fractions was assessed by Western blotting. Figure 3c shows that Neu3 treated either with Tris buffer or HAM remained associated with the membrane fraction and the same occurred with the control Cav-1. The effectiveness of the HAM treatment was evaluated by analyzing the behavior of ^N13^GAP43-YFP, a chimeric protein which binds to membranes via acylated cysteine residues at positions 3 and 4^31^. As shown in Fig. 3c, a fraction of membrane-bound GAP43 was released into the supernatant after treatment with HAM, indicating that this treatment was efficient for removing fatty acids from proteins. Thus, these experiments indicate that the covalently-bound fatty acid of Neu3 was not essential for its membrane association at steady state. Furthermore, since S-acylation is mostly restricted to the cytosolic side of the membranes^30^, the discovery of this post-translational modification strongly supports our idea that Neu3 has a portion exposed to the cytosol.

### Secondary structure prediction and homology modeling of Neu3

**Figure 4:**
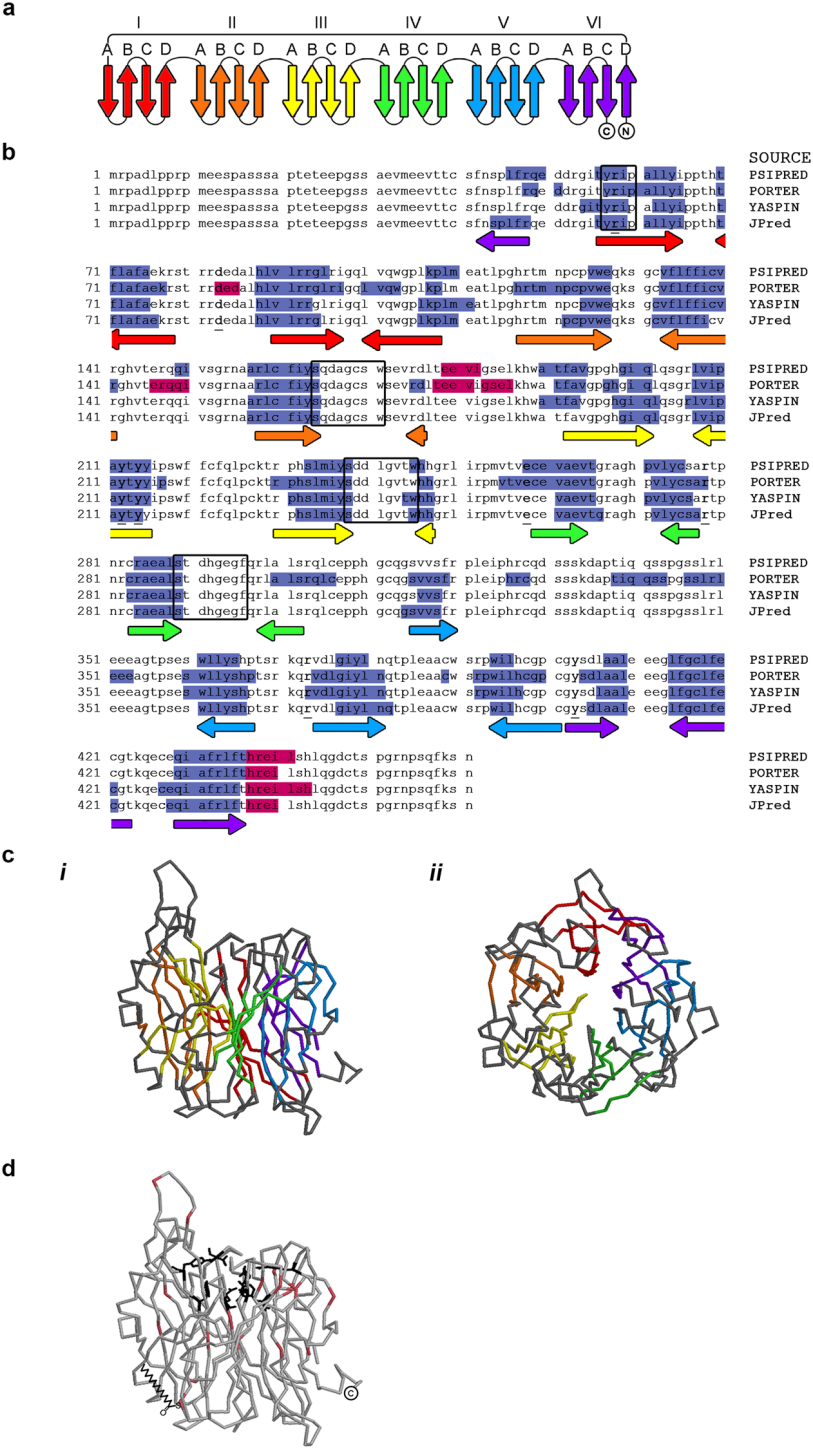
Secondary structure prediction and homology modeling of Neu3. (**a**) Common folding topology for the β-propeller domain of viral, bacterial and Neu2 sialidases. The fold consists of six (I-VI) four-stranded (A–D) antiparallel β-sheets (arrows) arranged as the blades of a propeller, with the N- and C-termini close to each other. (**b**) Prediction of secondary structure elements in human Neu3 sequence using different servers available on-line. Predicted β-sheets and α-helix are shown in blue and magenta boxes, respectively. Some of the highly conserved active site residues are shown in bold and underlined, whereas the conserved F/YRIP motif near the N-terminus and the three Asp boxes are shown in black boxes. The orientation of the predicted antiparallel β-sheets is shown with arrows. (**c**) Homology model of Neu3 using Neu2 crystal structure as a template. The β-sheets of the β-propeller backbone are shown in the same colors as in Fig. 4a and b. (*i*) Side view. (*ii*) Top view. (**d**) Schematic model of Neu3, highlighting in black sticks some residues from the active site. The C-terminus is indicated. Cysteins are labelled in pink and a hypothetical fatty acid is drawn attached to one of them.

All members of the sialidase family that have been crystallized, from virus and bacteria to mammals, have a common folding topology corresponding to the typical six-bladed β-propeller structure^18–20^ and consisting of six four-stranded antiparallel β-sheet motifs arranged as in the blades of a propeller with the N- and C-termini close to each other (Fig. 4a). Secondary structure prediction performed on Neu3 sequence by different prediction servers revealed the presence of many β-strand regions, a characteristic common to all the sialidases (Fig. 4b). Conversely, a low content of α-helices was predicted. We next created a Neu3 homology model in the Swiss-Model workspace using human cytosolic Neu2 crystal structure as a template. Neu2 is the only human sialidase whose crystal structure is available to date^18^, with both sequences sharing 43% identity after alignment using ClustalOmega. The modeled structure of Neu3 consisted of a β-propeller with a high content of β-strand portions (Fig. 4c), as already reported^21^.

The active site residues are also conserved in the sialidase family and are distributed throughout the sequence (in the case of Neu3, underlined in Fig. 4b). In the three-dimensional folded structure, the active site is found in the center of the β-propeller and on the top of the molecule. Several authors have modeled the ligand-bound conformation of Neu3 and identified the putative active site residues that are in contact with the substrate^21^, with some of those models having been validated through site directed mutagenesis^32, 33^. Although the putative active site residues vary from one study to another, some of them appear most frequently and are highly conserved among sialidases: a tyrosine residue (Tyr^403^) acting as the nucleophile, and an arginine triad (Arg^58^, Arg^278^ and Arg^373^), interact with the carboxylic group common to all natural sialic acid derivatives; two tyrosine residues (Tyr^212^ and Tyr^214^) interact with the glycerol side-chain of sialic acids contributing to substrate recognition; and two acidic residues (Asp^83^ and Glu^258^) are essential for acid-base catalysis during hydrolysis. These conserved catalytic residues are depicted in Fig. 4d as black sticks.

In addition, there are two unique motifs highly conserved among the sialidase family^34^: the Y/FRIP motif (Tyr/Phe-Arg-Ile-Pro) and the Asp-box motif (Ser/Thr-X-Asp-X-Gly-X-X-Trp/Phe; where X represents any amino acid). The Y/FRIP motif is located near the N-terminal part and contains an arginine residue involved in the formation of the arginine triad at the active site, and in particular, in Neu3, the Y/FRIP motif contains Arg^58^ (Fig. 4b). The Asp-box motif is repeated up to five times along the sequences in all sialidases of non-viral origin and interestingly, these repeated sequences are always located at equivalent positions: at the turn between the third and the fourth β-strands of certain blades, and far from the active site. In Neu3 sequence, there are three Asp boxes located in the second, third, and fourth blades of the β-propeller (Fig. 4b). The functions of the highly conserved Asp-boxes still remain to be determined.

Finally, it is worth mentioning that, although this superfamily of enzymes share the same basic fold, there is a considerable variation between them, which encourages the structure-based design of selective inhibitors^35–37^.

### Neu3 interacts with itself via disulfide bridges and with other proteins

**Figure 5:**
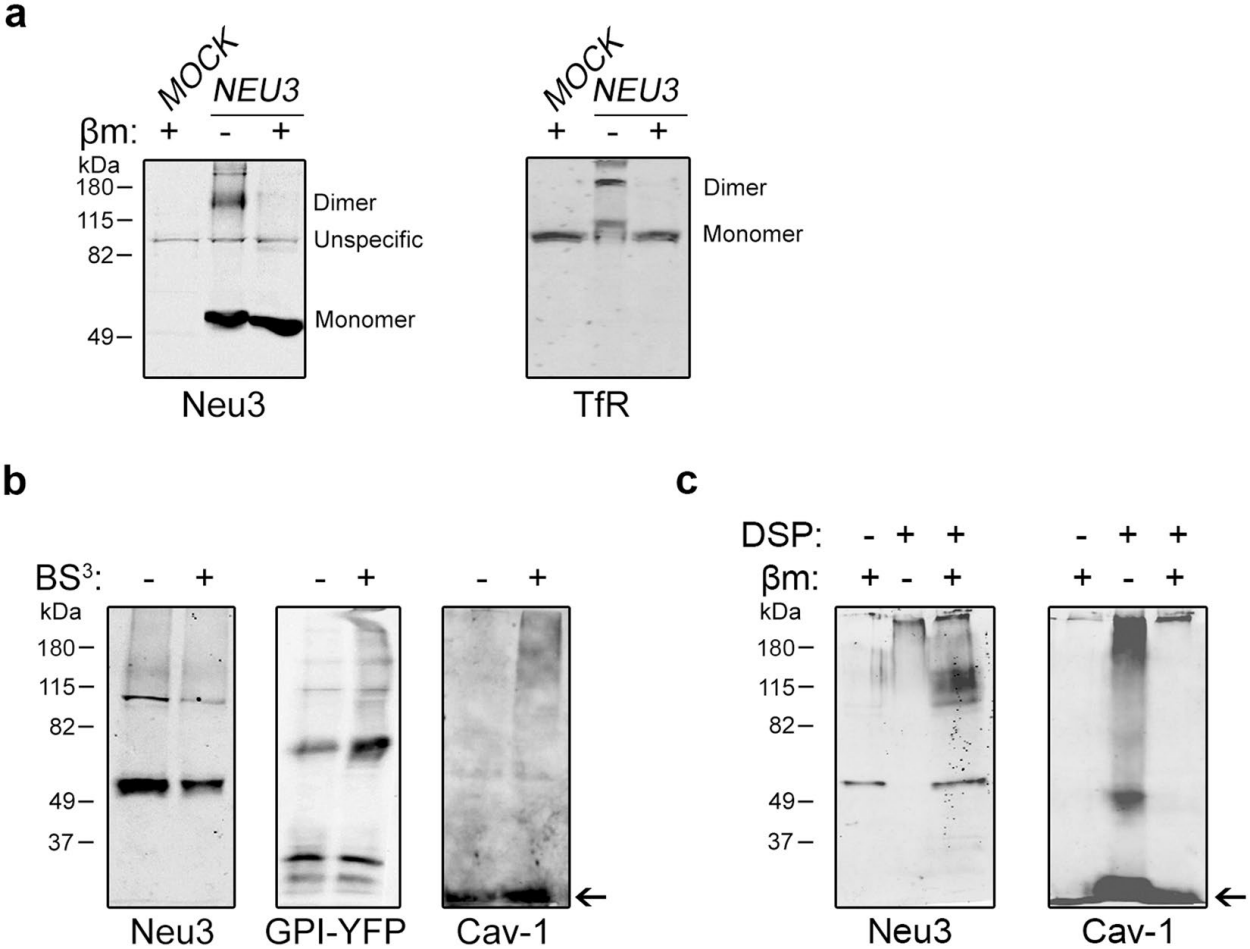
Neu3 interacts with itself via disulfide bridges and with other proteins. (**a**) Total homogenates of *MOCK*- and *NEU3*-transfected cells were separated by SDS-PAGE with (+) or without (−) 5% β-mercaptoethanol (βm), and Western blotted with antibody against Neu3 and antibody against endogenous TfR. (**b**) *NEU3*-transfected cells were subjected to chemical cross-linking with (+) or without (−) the membrane-impermeable reagent BS^3^. Then, proteins were separated by SDS-PAGE and detected by Western blotting using anti-Neu3 antibody. Cav-1 (indicated by an arrow) and GPI-YFP were used as markers of proteins not accessible and accessible to BS^3^, respectively. (**c**) *NEU3*-transfected cells were subjected to chemical cross-linking with (+) or without (−) the membrane-permeable reagent DSP. Then, proteins were separated by SDS-PAGE, and detected by Western blotting using anti-Neu3 antibody. Cav-1 (indicated by an arrow) was used as marker of a protein accessible to DSP. Due to the presence of a disulfide bridge in DSP molecule, the crosslinking can be reversed by the addition of βm. The molecular masses in kDa of the standard proteins are shown on the left.

Next, we attempted to address whether Neu3 could interact with itself and/or with other proteins. First, we analyzed by Western blotting the distribution of Neu3 under reducing and non-reducing conditions (Fig. 5a). In gels run with β-mercaptoethanol, a major band migrating at ~56 kDa was observed, as expected. We also observed a minor band at ~100 kDa in *NEU3*-transfected cells as well as in *MOCK*-transfected cells, indicating that this was an unspecific band detected by the antibody. On the other hand, in gels run in the absence of β-mercaptoethanol, a band migrating at approximately 125 kDa appeared, which accounted for 25% of the total enzyme. This result strongly suggested that the 125 kDa form was probably formed by a dimeric association via disulfide bridges of the monomeric 56 kDa form. TfR was used as an endogenous control of a known protein composed of two monomers (~95 kDa each) joined by disulfide bonds.

To study the association of Neu3 on the plasma membrane with itself or other molecules, we used chemical cross-linking with the membrane-impermeable agent BS^3^, which possesses a spacer arm of 1.2 nm. *NEU3*-transfected cells subjected to BS^3^ treatment showed no significant changes in the relative proportion among bands, indicating a low cross-linking efficiency of extracellularly exposed portion of Neu3 (Fig. 5b). A similar result was obtained for Cav-1, as expected for a protein not accessible to BS^3^. In contrast, when cells transiently expressing GPI-YFP (~37 kDa, monomer) were subjected to BS^3^ treatment, we observed a noticeable increase in the band of approximately 75 kDa (dimer), and also the appearance of bands at high molecular masses (multimers), as previously reported^22^ (Fig. 5b).

We then performed similar cross-linking experiments with the membrane-permeable reagent DSP, which possesses a spacer arm of 1.2 nm but, in contrast with BS^3^, it contains a disulfide bond in the spacer arm, which can be cleaved under reducing conditions. After DSP treatment, Neu3 was detected only as a high molecular weight complex (higher than 260 kDa), which hardly entered the running gel. However, cross-linking reversion, obtained by incubation of the DSP treated sample with β-mercaptoethanol, led to the reappearance of the Neu3 monomer band (Fig. 5c). When the same experiments were carried out to analyze the behavior of Cav-1 (~22 kDa, monomer), we observed that after DSP treatment a band of approximately 45 kDa (dimer) appeared together with bands at high molecular masses. Again, cross-linking reversion led to the reappearance of the band corresponding to Cav-1 monomer. Taken together, these experiments suggest that Neu3 has the ability to interact with other proteins and, particularly, with itself via disulfide bridges.

## Discussion

The present study provides a comprehensive analysis of Neu3 topology and brings new insights into its membrane-anchoring mechanism. We first confirmed that Neu3 was a membrane-bound protein associated with the plasma membrane and endosomes. However, we were unable to release the sialidase from membranes under conditions that typically release peripheral membrane proteins (Fig. 2a). Protease protection assays and antibody accessibility in non-permeabilized cells, suggested that the C-terminus of Neu3 was exposed to the cytosol (Fig. 2b and c). Related to this, since all sialidases have the N- and C-termini close to each other, it is probable that the N-terminus of Neu3 may be also exposed to the cytosol, although this remains to be confirmed. In addition, a portion of Neu3 polypeptide bound to the plasma membrane was accessible to PK and to cell-surface biotinylation, hence being exposed to the extracellular environment (Fig. 2b and d). Taken together, these experiments demonstrate that Neu3 behaves as an integral membrane protein that spans the entirety of the biological membrane to which it is attached. However, analysis of Neu3 sequence indicated that the protein does not contain any membrane-spanning hydrophobic α-helices that would justify our observations (Figs 2e and 4b), suggesting that the sialidase might span the lipid bilayer by a different mechanism. Homology modeling of human Neu3 using Neu2 crystal structure as the template, predicted that Neu3 is a typical six-bladed β-propeller composed of four antiparallel β-strands in each blade (Fig. 4c).

Importantly, we have shown that Neu3 is S-acylated (Fig. 3). Since this post-translational modification is mostly restricted to the cytosolic side of the membranes^28, 30^, it is likely that the acylated cysteine/s reside on the cytosolic-exposed portion of Neu3, strongly supporting the idea that Neu3 contains a cytosolic domain. Nevertheless, further studies are still needed to address which cysteines are being acylated, as well as to determine the biological implication of this post-translational modification. Due to S-acylation being distinct from other lipid modifications with respect to its reversibility and its dynamic regulation, we speculate that it could be important to determine the subcellular localization of the protein and to regulate its intracellular trafficking and its life span.

It has been recently described that Neu3 possesses the ability to interact with specific proteins at the plasma membrane and endosomes, with Neu3 interactors being identified by mass spectrometry and some of these being also confirmed by cross-immunoprecipitation with the enzyme^17^. Among the proteins identified that interact with Neu3 are flotillin-1, flotillin-2, clathrin heavy chain-1 (CLH1), sortin nexin-9 (SNX9) and Sec23A/Sec23B. Flotillins are strongly associated with the inner leaflet of the plasma membrane in a topologically manner similar to that of caveolins^38^. CLH1 and SNX9 are found in the cytosolic face of coated pits and vesicles, and are involved in endocytosis and intracellular vesicle trafficking^39^, whereas Sec23A/Sec23B are components of the COPII coat machinery, which recycle between a cytosolic and a membrane-bound pool^40^. In addition, it was also reported that Neu3 interacts with molecules such as Cav-1 and Grb2^11, 12^, and modulates clathrin-mediated endocytosis, probably by affecting the structural organization and subcellular distribution of the clathrin adaptor AP-2 complex^9^. Considering these above findings, how is it possible that a peripheral protein exposed to the extracellular space could interact with all these cytosolic or cytosolic membrane-bound proteins? Furthermore, there is evidence that Neu3 interacts with phosphatidic acid, a phospholipid mostly found in the inner-leaflet of membranes^41^. Our proposed membrane association of Neu3, compatible with a transmembrane protein, allows these interactions to be topologically favorable. Moreover, in line with these previous reports, we showed that Neu3 can interact with other proteins, since the sialidase could be cross-linked using a membrane-permeable reagent (Fig. 5c). As we did not observe protein cross-linking using a membrane-impermeable reagent (Fig. 5b), a premature speculation would be that Neu3 does not interact with extracytosolic domains of proteins. However, cross-linking experiments could be subjected to particularities of the reagent used, and therefore we cannot rule out that interaction of Neu3 with other extracellular components may actually exist. Another important fact that emerges from our experiments is that Neu3 is able to form homodimers, presumably joined by disulfide bonds (Fig. 5a). To our knowledge, this is the first description of Neu3 dimerization ability.

Taken into consideration most of the results obtained in this study, it is possible to hypothesize a model in which the whole β-propeller structure of Neu3 is inserted into the lipid bilayer. In this configuration, the portion of Neu3 having the catalytic region would be facing the extracellular environment (Fig. 4d), thus allowing its interaction with ganglioside substrates inserted in the outer leaflet of the plasma membrane or, eventually, with sialic acid associated to *N*-linked oligosaccharides of proteins. This would be also consistent with the fact that Neu3 is able to remove sialic acid residues from gangliosides present on adjacent cells^23^. Moreover, the opposite side of the catalytic crevice would be oriented towards the cytosol and would probably contain the acylatable cysteine/s and the C-terminus of the protein (Fig. 4d). However, this topological membrane configuration does not seem to be compatible with the fact that Neu3 may contain several charged amino acid residues that would be energetically unfavorable in a hydrophobic environment. This hypothetical inconsistency could be compensated by dimerization and/or association of the sialidase with other proteins, masking the charged amino acids of Neu3 that are exposed to lipids. Further studies are required to confirm these assumptions.

It is important to mention that some of our results are consistent with those obtained by Miyagi *et al*.^3^ who proposed that Neu3 was a type I integral membrane protein with its C-terminus facing the cytosol. However, it is now known that this topology does not allow the formation of the β-propeller. Interestingly, a recent study demonstrated that human sialidase Neu1 also behaves as a transmembrane protein with its N- and C-termini oriented towards the cytosol and with dimerization ability^42^, which is in sharp line with most of the results obtained in this study for Neu3.

The mechanisms that regulate Neu3 folding and its transport to the cell membranes have yet to be elucidated. It is known that most bacterial sialidases are secreted proteins that contain signal peptides which are cleaved during the protein secretion process^34^. In the human Neu3 sequence, however, no *N*-terminal cleavable signal peptide was predicted using SignalP 4.1 server (http://www.cbs.dtu.dk/services/SignalP/)^43^. Moreover, no *N*-glycosylation sites were predicted using NetNGlyc 1.0 server (http://www.cbs.dtu.dk/services/NetNGlyc/), and we further observed that the presence of tunicamycin, an en bloc *N*-glycosylation inhibitor, resulted in no change in the mobility of the protein on Western blots (results not shown), indicating low probability of Neu3 having *N*-glycans in its structure. Altogether, these results allowed us to speculate that Neu3 probably does not enter the secretory pathway, indicating a possible non-conventional mechanism of transport and insertion into the plasma membrane.

In conclusion, our study provides several experimental evidences which prove that Neu3 has the characteristics of an integral membrane protein, although it remains to be determined exactly how a predicted β-propeller can span the lipid bilayer in the absence of any easily detectable transmembrane segment. Further clarification of the topology of Neu3 could be achieved by other techniques such as crystallography, immunoelectron microscopy and super-resolution microscopy, in combination with computational approaches. This information is critical for better understanding the regulatory mechanisms of this enzyme at a molecular level and its participation in numerous biological processes.

## Methods

### Cell culture and transfection

CHO-K1 cells (A.T.C.C., Manassas, VA, USA) were cultured in Dulbecco’s modified Eagle’s medium (Invitrogen, Carlsbad, CA, USA) containing 10% (v/v) fetal bovine serum and antibiotics (100 µg/ml penicillin and 100 µg/ml streptomycin), and were maintained at 37 °C and 5% CO_2_ in a humidified incubator. Transfections were carried out using polyethylenimine (Sigma-Aldrich, St Louis, MO, USA) in a serum-free medium with 1 μg of the indicated plasmid per 35 mm-diameter-dish, for 3 h at 37 °C. Cells were transiently transfected with a C-terminal c-Myc-tagged form of human Neu3 (NCBI Reference Sequence: NP_006647.3) synthesized by Genscript (Piscataway, NJ, USA), and subcloned into the pCI-neo mammalian expression vector (Promega, Madison, WI, USA). C-terminal HA-tagged human Neu3 was amplified by PCR using Neu3 cDNA as template and subcloned into the pCI-neo vector. Expression plasmids for YFP-K-Ras^C14^, GPI-YFP, ST3Gal-II-HA and ^N13^GAP43-YFP described previously^22, 24, 44, 45^ were used as controls in the indicated experiments. Cells were analyzed at 24 h post-transfection.

### Antibodies

The following antibodies were used: monoclonal mouse antibody against human Neu3 (MBL International Corporation, Woburn, MA, USA), monoclonal mouse antibody against c-Myc epitope (Sigma-Aldrich, St Louis, MO, USA), polyclonal rabbit antibody against c-Myc epitope (Sigma-Aldrich, St Louis, MO, USA), monoclonal mouse antibody against ganglioside GD3 (clone R24; A.T.C.C., Manassas, VA, USA), monoclonal mouse antibody against ganglioside GD1a (kindly supplied by P.H. Lopez, INIMEC-CONICET, Córdoba, Argentina), polyclonal rabbit antibody against caveolin-1 (Abcam, Cambridge, UK), monoclonal mouse antibody against α-tubulin (Sigma-Aldrich, St Louis, MO, USA), monoclonal mouse antibody against GFP (Roche Diagnostics, Basel, Switzerland), monoclonal mouse antibody against transferrin receptor (Invitrogen, Carlsbad, CA, USA), monoclonal mouse antibody against HA epitope (Sigma-Aldrich, St Louis, MO, USA) and polyclonal rabbit antibody against HA epitope (Sigma-Aldrich, St Louis, MO, USA). The secondary antibodies used were goat anti-rabbit- or mouse-IgG coupled to Alexa Fluor 488 or Alexa Fluor 546 (Invitrogen, Carlsbad, CA, USA) for immunofluorescence, and goat anti-rabbit- or mouse-IgG coupled to IRDye800CW or IRDye680RD (LI-COR Biotechnology, Lincoln, NE, USA) for Western blotting.

### Immunofluorescence staining and confocal microscopy analysis

Cells grown on glass coverslips were washed twice with PBS and fixed with 1% (w/v) paraformaldehyde in PBS for 10 min at room temperature. After three washes with PBS, cells were permeabilized with 0.1% saponin (Sigma-Aldrich, St Louis, MO, USA) in PBS containing 1% (w/v) BSA (PBS/BSA) for 10 min at room temperature. Then, cells were washed and incubated overnight at 4 °C with primary antibodies diluted in PBS/BSA. Finally, cells were washed and incubated for 2 h at 37 °C with secondary antibodies diluted in PBS/BSA. For immunofluorescence of non-permeabilized cells, the same procedure was used, but substituting PBS/BSA for 0.1% saponin. Nuclei were stained blue with Hoechst 33258 dye (Molecular Probes, Eugene, OR, USA). For staining of the plasma membrane-associated GD3, GD1a and GM1 gangliosides, cells were incubated with antibody against GD3, antibody against GD1a or Cholera Toxin β-subunit (CTx*β*) coupled to Alexa Fluor 555 (Molecular Probes, Eugene, OR, USA), respectively, at 4 °C for 60 min before fixation and permeabilization. Confocal images were collected using an Olympus FluoView FV1200 confocal microscope (Tokyo, Japan). Single confocal sections of 0.8 μm were taken parallel to the coverslip (xy sections). Final images were compiled with Adobe Photoshop CC.

### Sialidase enzymatic assays

The enzymatic activity of total sialidases in cell lysates was determined using 4MU-NeuAc (2-(4-methylumbelliferyl)-*α*-D-*N*-acetylneuraminic acid) (Sigma-Aldrich, St Louis, MO, USA) as substrate. Reactions were set up in triplicate in a final volume of 200 μl with 30 µg of proteins, 0.2 mM 4MU-NeuAc and 0.5 mM BSA in buffer citrate at pH 3.5. Reaction mixtures were incubated at 37 °C for 1 h and were stopped by the addition of 0.25 M glycine at pH 10. Fluorescence emission was measured on a FluoroMax-P spectrofluorometer (Horiba Jobin Yvon, Edison, NJ, USA) with excitation at 365 nm and emission at 445 nm.

### Western blotting

Proteins were separated by SDS-PAGE under reducing or non-reducing conditions and transferred onto nitrocellulose membranes (GE Healthcare, Little Chalfont, UK). Membranes were then blocked with 5% (w/v) defatted dried milk in 200 mM NaCl and 50 mM Tris/HCl pH 7.5 (TBS), and incubated overnight at 4 °C with primary antibodies diluted in TBS containing 0.05% Tween 20 (TTBS). After three washes with TTBS, membranes were incubated with secondary antibodies diluted in TTBS for 2 h at room temperature. Bands of proteins were detected using an Odyssey infrared imaging system (LI-COR Biotechnology, Lincoln, NE, USA). Molecular masses were calculated based on calibrated standards run in parallel.

### Subcellular fractionation

Cells were washed with cold PBS and harvested by scraping in 5 mM Tris/HCl pH 7.0 (buffer T) supplemented with protease inhibitor cocktail (PIC) (Sigma-Aldrich, St Louis, MO, USA). Extracts were centrifuged at 4 °C for 5 min at 10,000 *g* and resuspended in buffer T-PIC. Pellets were dispersed by vortex every 10 min during an hour, and passed 60 times through a 25-gauge needle. Nuclear fractions and unbroken cells were removed by centrifugation at 4 °C for 5 min at 500 *g*. Homogenates were then ultracentrifuged at 4 °C for 1 h at 400,000 *g* using a TLA 120.1 rotor (Beckman Coulter, Brea, CA, USA). The supernatant fraction (S) was collected, and the pellet fraction (P) was resuspended in buffer T-PIC. The proteins in both fractions were precipitated with chloroform:methanol (1:4 v/v) or used for subsequent analysis.

### Membrane protein extraction

The cell membrane fraction (P) obtained after subcellular fractionation was resuspended in buffer T-PIC and split into equal aliquots. Extraction of peripheral membrane proteins was performed by exposure of the P fraction to 0.1 M Na_2_CO_3_ (Sigma-Aldrich, St Louis, MO, USA) at pH 11.5, followed by incubation at 4 °C for 40 min. For deacylation of membrane proteins, the P fraction was resuspended in freshly prepared 1 M hydroxylamine (Sigma-Aldrich, St Louis, MO, USA) titrated to pH 8 with NaOH, and incubated for 1 h at room temperature. In the case of control samples, membranes were incubated in the presence of buffer T-PIC alone. Finally, extractable and non-extractable proteins were separated by ultracentrifugation at 400,000 *g* for 1 h at 4 °C, and soluble and membrane fractions were adjusted to the same final volume, precipitated with chloroform:methanol (1:4 v/v) and analyzed by Western blotting.

### Proteinase K protection assays

Intact cells were incubated with 100 µg/ml Proteinase K (Sigma-Aldrich, St Louis, MO, USA) for 30 min at 37 °C. Then, cells were washed with PBS, collected, lysed and processed for Western blotting analysis.

### Cell-surface protein biotinylation

Intact cells were labelled with 0.5 µg/µl of membrane-impermeable EZ-Link Sulfo-NHS-SS-biotin (Pierce Biotechnology, Waltham, MA, USA) in PBS for 1 h at 4 °C. After being washed three times with PBS and the free biotin quenched by the addition of 50 mM glycine for 15 min at 4 °C, cells were scraped and treated with lysis buffer (20 mM Tris/HCl pH 7.0, 1 mM EDTA, 1% Triton X-100, 150 mM NaCl, 10 mM glycine and supplemented with PIC) for 1 h at 4 °C with gentle agitation. After lysis, nuclear fractions and unbroken cells were removed by centrifugation at 4 °C for 5 min at 500 *g*, and biotinylated proteins were separated from non-biotinylated proteins using streptavidin agarose beads (Pierce Biotechnology, Waltham, MA, USA). Fractions were then adjusted to the same final volume, precipitated with chloroform:methanol (1:4 v/v) and analyzed by Western blotting.

### Acyl-Biotin Exchange (ABE)

The Acyl-Biotin Exchange was carried out as described in ref. 46 with some modifications. Briefly, proteins from total cell homogenates were precipitated using chloroform:methanol (1:4 v/v) and treated overnight at 4 °C with 3 mM N-ethylmaleimide (NEM) (Sigma-Aldrich, St Louis, MO, USA) to block the free thiol groups. Free NEM was then removed by three sequential chloroform:methanol (1:4 v/v) precipitations, and samples were treated with 1 M hydroxylamine (Sigma-Aldrich, St Louis, MO, USA) and 1 mM EZ-Link HPDP-Biotin (Pierce Biotechnology, Waltham, MA, USA) for 1 h at room temperature to exchange thiol-bound fatty acids for biotin. In this step, omission of hydroxylamine served as a negative control. Samples were then incubated with streptavidin agarose beads (Pierce Biotechnology, Waltham, MA, USA) for 1 h at room temperature. All incubations were carried out in a buffer containing 50 mM Tris/HCl pH 7.0, 5 mM EDTA, 0.2% Triton X-100, 150 mM NaCl, 10 mM phenylmethanesulfonyl fluoride and PIC. Finally, all samples were centrifuged at 13,000 *g* for 1 min, unbound proteins were removed and the palmitoylated ones were eluted from the beads with 75 mM Dithiothreitol (DTT) for 10 min at 90 °C and processed for Western blotting. A commercial human Neu3 (Origene, Rockville, MD, USA) purified from HEK-293 cells was also subjected to ABE analysis.

### Cross-linking assays

Intact cells were incubated at 4 °C with 2 mM of membrane-impermeable bis(sulfosuccinimidyl)suberate (BS^3^) (Sigma-Aldrich, St Louis, MO, USA), or at room temperature with 0.5 mM of membrane-permeable dithiobis(succinimidyl propionate) (DSP) (Thermo Scientific, Waltham, MA, USA) for 45 min. Cross-linking was quenched by the addition of 50 mM glycine for 15 min at 4 °C. Cells were then washed with PBS, collected, lysed and processed for Western blotting analysis.

### Prediction of secondary structure and homology modeling

For the prediction of secondary structure elements in Neu3 sequence, different available on-line servers were used: PSIPRED (http://bioinf.cs.ucl.ac.uk/psipred/)^47^, PORTER (http://distill.ucd.ie/porter/)^48^, YASPIN (http://ibi.vu.nl/programs/yaspinwww/)^49^ and JPred (http://www.compbio.dundee.ac.uk/jpred/)^50^. Homology modeling of Neu3 in the Swiss-Model workspace (https://swissmodel.expasy.org)^51^ was performed in automatic mode using Neu2 crystal structure (PDB ID: 1SNT) as a template, and the model was visualized using RasMol software (Version 2.7.5.2).
